# Current potential biomarkers for Alzheimer’s disease, Parkinson’s disease and amyotrophic lateral sclerosis: review of literature

**DOI:** 10.1080/19585969.2026.2622722

**Published:** 2026-02-06

**Authors:** Jiaman Peng, Ting Fan, Junling Wang, Youqing Deng, Renshi Xu

**Affiliations:** a Jiangxi Medical College, Nanchang University, Nanchang, China; b Department of Neurology, Jiangxi Provincial People’s Hospital, The Clinical College of Nanchang Medical College, The First Affiliated Hospital of Nanchang Medical College, Xiangya Hospital of Center South University, Jiangxi Hospital, Nanchang, Jiangxi Province, China; c National Regional Center for Neurological Disease, Nanchang, Jiangxi Province, China; d Department of Neurology, The First Hospital of Nanchang, Nanchang, Jiangxi Province, China

**Keywords:** Neurodegenerative diseases, biomarkers, Alzheimer’s disease, Parkinson’s disease, amyotrophic lateral sclerosis

## Abstract

**Introduction:**

Alzheimer’s disease (AD), Parkinson’s disease (PD) and amyotrophic lateral sclerosis (ALS) are several common neurodegenerative diseases (NDs). At present, is the lack of effective diagnosis, progression, prognosis and therapeutic biomarkers. it is a urgent demand to search the relevant confident biomarkers.

**Area covered:**

This review systematically analysed the potential biomarkers of blood, cerebrospinal fluid, neuroimaing and emerging non-invasive indicators, and synthesises current evidences on the biomarkers of AD, PD and ALS about diagnosis, progression, prognosis and therapeutic, especially diagnosis biomarkers.

**Expert commentary:**

In this review, we focus on discussing relevant diagnosis, progression, prognosis and therapeutic biomarkers for AD, PD and ALS in recent years, and prospecting the possible future directions of relevant biomarkers.

## Introduction

Alzheimer’s disease (AD) is the most common neurodegenerative disease and the leading cause of dementia. AD is characterised by progressive memory impairment, declining cognitive functions, concomitant psychiatric and behavioural abnormalities. AD is a multifaceted disorder with complex pathological mechanisms that drives its decades-long chronic progression. Critical hallmarks include the accumulation of β-amyloid (Aβ) plaques and neurofibrillary tau tangles in brain, alongside neuronal loss and synaptic dysfunction. Based on recent statistics, there are approximately 5.8 million individuals with AD in United States, with prevalence surging to nearly 30% among adults aged 85 or older (Xu et al. 2021). AD is closely association with ageing and age-dependent increase, which brings significant challenges for healthcare systems globally following global ageing progression. As life expectancy continues to rise, the burden of AD is expected to grow exponentially, urgent need improve diagnostic tools for disease-modifying therapies and preventive strategies.

Parkinson’s disease (PD) is the second commonest neurodegenerative disease, primarily affecting motor functions. PD is characterised by bradykinesia (slowed movement), muscle rigidity, resting tremor and postural/gait abnormalities, PD results from the progressive loss of dopaminergic neurons in substantia nigra, leading to the deficiency of dopamine neurotransmitter in striatum. The neurodegenerative process of PD mainly impairs movement, and often progresses to non-motor symptoms such as cognitive decline, sleep disturbances and autonomic dysfunction. In United States, PD affects approximately 1 million individuals with prevalence increasing age. Although motor symptoms are the hallmark of PD, it also impacts extends beyond movement disorders, which need comprehensive, multidisciplinary management strategies. As with other neurodegenerative diseases (NDs), the early diagnosis of PD remains challenging, it is a critical need diagnostic biomarkers that can detect preclinical pathophysiological alterations and monitor progression (Xu et al. 2021).

Amyotrophic lateral sclerosis (ALS) is a fatal neurodegenerative disease characterised by the progressive loss of upper and lower motor neurons, leading to widespread voluntary muscle weakness, atrophy and eventual respiratory failure (Vidovic et al. 2023). ALS has a poor prognosis with a median survival time of 2 to 4 years, and limited options for aetiological treatment. Protein biomarkers for the diagnosis and prognosis of ALS has been made an inspiring progress, which is a promising objective auxiliary tool for diagnosing ALS (Soares Martins et al. 2021).

Among the common pathophysiologies of NDs, the deposition of misfolded and fragmented proteins in central nervous system (CNS), neuroinflammation, the dysregulation of glutamatergic signalling, oxidative stress and cytotoxic effects are the most prominent pathophysiologies, which are at some scales related to both neurological and neuronal damage (Donini et al. 2023). With ageing populations worldwide, the prevalence of NDs is gradually rising by year to year, imposing substantial economic and familial burdens. Despite intensive researches, curative interventions for AD, PD and ALS remain scarce. Early and accurate diagnosis is critical to optimise existing symptomatic treatments, enrol patients in clinical trials and facilitate the development of disease modifying therapies. Biomarker auxiliary diagnostic approaches integrating biofluid alteration analyses, neuroimaging and digital health technologies are hopeful to achieve this goal. To this end, this review summarised the current potential diagnosis, progression, prognosis and therapeutic biomarkers, especially diagnosis biomarker, aimed to provide some clues for further exploring diagnosis, progression, prognosis and therapeutic biomarkers, improving early accurate diagnosis, accurately assessing disease progression and prognosis, objectively evaluating drug therapeutic effects (Table 1).

**Table 1.** Summary of biomarkers for Alzheimer’s Disease (AD), Parkinson’s Disease (PD) and Amyotrophic Lateral Sclerosis (ALS).

Diseases	Biomarker	Association with Biomarker	References
AD	Aβ42	The aggregation of Aβ42 in brain forms amyloid plaques, leading to decrease its content in cerebrospinal fluid, is a potential diagnosis biomarker	(Soares Martins et al. 2021,Donini et al. 2023)
	t-tau/p-tau	After abnormal phosphorylation, tau protein forms neurofibrillary tangles, disrupting the normal function of neurons and increasing the content of related proteins in cerebrospinal fluid. t-tau/p-tau is potential diagnosis biomarkers.	(Kocurova et al. 2022,Ishigaki and Sobue 2018)
	Chi3l1/ YKL-40	Chi3l1/YKL-40 can activate microglia and astrocytes, promote the release of inflammatory factors and exacerbate neuroinflammatory responses. Chi3l1/YKL-40 is a potential diagnosis biomarker.	(Craig-Schapiro et al. 2010; Querol-Vilaseca et al. 2017; Zeng et al. 2023)
	NPTX	NPTX binds to receptors on the surface of neurons, affecting neuronal excitability and synaptic transmission efficacy, which influences the localisation and function of postsynaptic receptors, playing a role in the structural and functional plasticity of synapses. NPTX is a potential diagnosis biomarker.	(Gómez De San José et al. 2022; Dejanovic et al. 2018; Dejanovic et al. 2022)
	miRNA	The concentration of miRNA-125b in the serum of AD patients is decreased, indicating its potential as a biomarker for AD.	(Wang and Zhang 2020; Gaetani et al. 2020; Long et al. 2025; Smith et al. 2015; Li et al. 2024; Liu et al. 2014)
	NF-L	The elevation of NF-L levels in blood can reflect the degree of neuronal axonal damage and is associated with the progression of AD, is important progression biomarker of AD.	(Arslan et al. 2024; Monteiro et al. 2023; Hansson et al. 2023)
	MRI	MRI can visually demonstrate the condition of brain atrophy, is potential diagnosis, progression biomarker of AD.	(Dang et al. 2023)
**AD**	MRS	MRS measures neurochemical substances and the concentration or ratio of their metabolites, can serve as biomarkers for AD diagnosis biomarker.	(Femminella et al. 2018)
	PET	PET can measure metabolic changes with the aid of radioactive tracers and plays an important role in the diagnosis and prognostic evaluation of AD as biomarker candidate.	(Femminella et al. 2018)
	SPECT	SPECT can detect changes in cerebral blood flow and metabolism, which is of great significance in the diagnosis of AD.	(Prasath and Sumathi 2023)
	FDG-PET	FDG-PET servers as a diagnosis biomarker by detecting changes in glucose metabolism.	(Casaletto et al. 2017)
	Retinal imaging technology	Retinal imaging technology can deeply analyse the structural and functional changes of retina through technical means such as image reconstruction, filtering processing, image segmentation and image registration. Retinal alteration may sever as helpful diagnosis biomarker.	(Casaletto et al. 2017)
	Saliva	The concentration of lactoferrin in saliva is reduced in patients with AD and mild cognitive impairment, making it useful for early disease diagnosis biomarker. The activity of acetylcholinesterase in saliva is also decreased, and this reduction is drug response biomarker.	(Ashton et al. 2019)
	Saliva	Acetylcholinesterase level in saliva is also decreased, and this reduction is drug response biomarker.	(Kaštelan et al. 2023)
**AD**	Urine Tears	Substances such as 8-hydroxydeoxyguanosine (8-OHdG) and a subtype of neuron-specific enolase (AD7c-NTP) in urine, as well as some proteins in tears, may all serve as potential diagnosis biomarkers for AD, although further in-depth research is still needed.	(Majeed et al. 2021)
	Retina	Changes in β-amyloid (Aβ) plaques in retina, alterations in the thickness of the retinal nerve fibre layer (RNFL), and modifications in retinal vascular parameters are potential diagnosis biomarker of AD.	(Villa et al. 2020)
**PD**	α-Synuclein	In patients with Parkinson’s disease (PD), the levels of α-synuclein oligomers in cerebrospinal fluid may increase. The Lewy bodies formed by their aggregation are important pathological features of PD, which can reflect the pathological changes within neurons. α-synuclein oligomers is an important diagnosis biomarker for PD.	(Sechi and Sechi 2024,Ciechanover and Kwon 2015)
	NF-L	NF-L is associated with disease severity and progression. Elevated NF-L levels in cerebrospinal fluid indicate neuronal axonal injury. NF-L is an important PD severity and progression biomarker.	(Van Der Zee et al. 2021)
	PARK7/DJ-1	PD protein 7 (PARK7/DJ-1) regulates the expression and function of molecular chaperone proteins, influencing the folding and transport processes of α-synuclein. PARK7/DJ-1 promotes α-synuclein misfolding and aggregation, leading to the formation of Lewy bodies and impairing neuronal function. PARK7/DJ-1 is a potential diagnosis biomarker candidate.	(Lockhart et al. 2004; Morrone Parfitt et al. 2024; Jun and Kool 2020; Wei et al. 2018; Choi et al. 2019; Hasim et al. 2014; Tong et al. 2015; Tsunemi et al. 2020; Streubel-Gallasch et al. 2021; di Domenico et al. 2019; Wilson et al. 2019)
**PD**	GCase	The lysosomal enzyme glucocerebrosidase (GCase) plays a key role in the pathogenesis and progression of PD by participating in α-synuclein metabolism, maintaining lysosomal function, influencing mitochondrial function, and regulating inflammatory responses. GCase is a candidate diagnosis biomarker.	(Zhang et al. 2024)
	PET/SPECT	Dopaminergic positron emission tomography (PET)/single-photon emission computed tomography (SPECT) can detect abnormalities in dopaminergic system function and the loss of dopamine innervation. Thus, these two PET and SPECT clinical examination are PD important diagnosis biomarkers.	(Biondetti et al. 2020)
	Non-dopaminergic PET	Non-dopaminergic PET imaging techniques cover the detection of serotonergic and cholinergic systems, as well as neuroinflammation-related biomarkers. Although they exhibit certain sensitivity to PD states, their application potential as early diagnostic biomarkers for PD still requires further clarification.	(Van Der Zee et al. 2021)
	Diffusion Imaging Techniques	At the early stages of PD, the increased free water content in the posterior substantia nigra makes it a promising biomarker for reflecting disease status and progression.	(Gaurav et al. 2021)
	MRI	Neuromelanin-sensitive magnetic resonance imaging can observe the reduction in neuromelanin signal intensity in the substantia nigra region, suggesting be potential biomarker in evaluating PD disease status during the prodromal phase.	(Gaurav et al. 2021)
**PD**	Iron-sensitive MRI technique	The R2* parameter may have higher sensitivity to disease progression, is a promising biomarker candidate assessing PD progression.	(Mitchell et al. 2021)
	Automatic speech analysis	Although there are phonetic differences between different languages, the overall trends in the relationship between automatic speech analysis and condition assessment are the same. Automatic speech analysis is a useful biomarker assessing disease statues of PD.	(Rusz et al. 2021)
**ALS**	TDP-43	In ALS patients, TDP-43 misfolds and transfers from the nucleus to the cytoplasm to form aggregates, interfering with normal cellular functions. As an RNA-binding protein, its dysfunction leads to RNA metabolic disorders. Aggregates in the cytoplasm also activate stress signalling pathways, disrupt cytoskeletal functions, and generate neurotoxicity. Additionally, mutations in the TDP-43 gene increase risk developing ALS. TDP-43 is a genetics diagnosis biomarker of ALS.	(Sreedharan et al. 2008; DeJesus-Hernandez et al. 2011; Bonafede and Mariotti 2017; Ferrara et al. 2018; Prasad et al. 2019; Silverman et al. 2019; Suk and Rousseaux 2020; Yuan et al. 2021; Hayes and Kalab 2022)
	SOD1	SOD1 mutants aggregate in extracellular vesicles, which exhibits the prion-like propagation and spread of ALS pathology in central nervous system. Thus, SOD1 mutants is a genetics diagnosis biomarker.	(Tiwari et al. 2005; Bonafede and Mariotti 2017; Fetherolf et al. 2017; Vu and Bowser 2017; Ferrara et al. 2018; Vejux et al. 2018; Huai and Zhang 2019; Mathis et al. 2019; Morimoto et al. 2019; Silverman et al. 2019; Yuan et al. 2021; Darabi et al. 2024)
	C9orf72	The hexanucleotide repeat expansion mutation in C9orf72 may lead to increased toxicity of C9orf72, resulting in loss of its function, which indicates that hexanucleotide repeat expansion mutation in C9orf72 is a genetics diagnosis biomarker of ALS.	(Mizielinska et al. 2025)
	miRNA	miRNA plays an important role in multiple pathophysiological processes of ALS, such as neuronal survival, neuroinflammatory response and maintenance of neuromuscular junction function by regulating gene expression. Therefore, miRNAs are an important genetics diagnosis biomarkers of ALS.	(Freischmidt et al. 2015; Waller et al. 2017; Rajgor 2018; Ricci et al. 2018; Ravnik-Glavač and Glavač 2020; Dobrowolny et al. 2021; Lee and Woodruff 2021)
**ALS**	NF-L/pNF-H	Released upon neuronal injury, the elevated blood levels of NF-L/pNF-H are associated with ALS severity and prognosis, making it the most promising diagnostic, severity assessment and prognostic biomarker to date.	(Brettschneider et al. 2006; Boylan et al. 2013; Ganesalingam et al. 2013; Lu et al. 2015; Bacioglu et al. 2016; Gendron et al. 2017; Ishigaki and Sobue 2018; Poesen and Van Damme 2018; Benatar et al. 2020; Miteva et al. 2023)
	IL-6, TNF-α	Inflammatory cytokines such as IL-6 and TNF-α reflect neuroinflammation but have low specificity, which may overlap with other inflammatory diseases, is a reference diagnosis biomarker.	(Ganesalingam et al. 2013; De Paula Martins et al. 2018; Brodovitch et al. 2021; Shepheard et al. 2022)
	Cystatin C	Cystatin C protects motor neurons from damage by stimulating autophagy and inhibiting the neurotoxicity of cathepsin B, is a promising diagnosis biomarker.	(Watanabe et al. 2014,Zhu et al. 2018)
	TTR	In ALS patients, transthyretin (TTR) continues to decline, and the decrease in TTR levels in ALS spinal cord may be due to a reduction in the number of motor neurons and a decrease in TTR expression levels in the remaining motor neurons. TTR is progression and prognosis biomarker candidate of ALS.	(Watanabe et al. 2014)
	CRP	Serum CRP levels exhibit a significant negative correlation with the ALSFRS-R score of ALS, suggesting that serum CRP levels are an important biomarker in assessing the utility, feasibility and prognosis of ALS.	(Darabi et al. 2024)
	CK, UA	Elevated CK indicates muscle damage, is an assisted diagnosis biomarker. Uric acid may be associated with disease progression, is an assisted progression assessment biomarker, but their specificity is limited.	(Keizman et al. 2009,Chaves-Filho et al. 2019)
	CHIT1	The level of CHIT1 may reflect the activation degree of microglia/macrophages in the spinal cord white matter. CHI1 may be a potentially useful biomarker for the differential diagnosis of ALS and prediction of disease progression.	(Yang et al. 2021)
**ALS**	Lipid	Abnormal lipid metabolism is closely associated with neurodegenerative diseases, among which bismonoacylglycerol phosphate (BMP) and glycosphingolipids such as gangliosides GM1, GD1a and glucocerebroside GlcCer) are the most promising diagnosis biomarker candidates for ALS.	(Guo et al. 2024,Wei et al. 2023)
	Gut microbiota	Gut microbiota influences the progression of ALS through immune regulation, neuroinflammation, and metabolites such as short-chain fatty acids. Dysbiosis may exacerbate motor neuron damage, while microbiota regulation could become a potential therapeutic biomarker.	(Darabi et al. 2024)
	Metabolic hormones	Metabolic hormones such as insulin and amylin may exert effects by regulating energy metabolism, neuroinflammation and neuronal protective mechanisms. Metabolic hormones may become a assisted diagnosis and therapeutic biomarker.	(Moțățăianu et al. 2024)

## Alzheimer’s disease

### The pathophysiologies of AD

The accumulation of Aβ plaques and tau protein neurofibrillary tangles represents two cardinal pathological hallmarks in AD pathophysiology. Neuroinflammation mediated by glial cells and subsequent neuronal loss are strongly correlated with cognitive impairment in AD, accompanying with molecular mechanisms encompassing immunological dysregulation, inflammatory responses, oxidative stress, vascular dysfunction and excitotoxicity (Soares Martins et al. 2021). Aβ, the primary component of extracellular plaques in AD, originates from transmembrane amyloid precursor protein (APP), which is widely expressed across multiple tissues and preferentially localised in synaptic regions. APP processing may facilitate the generation of Aβ variants, among them, Aβ140 is recognised as a highly neurotoxic species (Kocurova et al. 2022). Based on amyloid cascade hypothesis, Aβ pathology is postulated to initiate a downstream cascade driving the formation of tau neurofibrillary tangles and progressive neurodegeneration in AD (Donini et al. 2023).

Tau, a naturally unfolded microtubule-associated protein, is predominantly expressed in axons, where it plays a critical role in regulating axonal transport processes (Ishigaki and Sobue 2018; Kocurova et al. 2022). Pathological tau undergoes intercellular transmission, triggering the conversion of normal tau into misfolded conformations and promoting the formation of tau aggregates in neurons (Ishigaki and Sobue 2018). In the pathophysiology of AD, specific post-translational modifications like phosphorylation in proline-rich regions accelerate aggregated tau into filamentous structures (Wesseling et al. 2020). Mutations in microtubule-associated protein tau (MAPT) gene are linked to widespread tauopathies including AD, frontotemporal dementia and memory impairment. Exon 10 of MAPT encodes microtubule-binding domain, which generates two major tau subtypes: 3-repeat (3 R) and 4-repeat (4 R) tau (Ishigaki and Sobue 2018; Rösler et al. 2019; Wesseling et al. 2020; Kocurova et al. 2022; Donini et al. 2023). Pathogenic MAPT mutations reduce the affinity of microtubule-binding domain for microtubules, disrupting the formation of stable 3 R/4R tau-microtubule complexes, which leads to a gradual increase in free tau concentration beyond a critical threshold, prompting misfolded tau to self-assemble into toxic oligomers, protofibrils and the ultimate neurofibrillary tangles-key drivers of neurodegeneration in AD (Campese et al. 2021).

Based on the current researches, tau proteins are clinically served as the robust biomarkers of AD progression. The cerebrospinal fluid (CSF) levels of phosphorylated tau (P-tau) reflect the extent of tau phosphorylation and neurofibrillary tangles burden in brain, while total tau (T-tau) correlates with the severity of neurodegeneration and neuronal damage in AD pathogenesis.

### The CSF biomarkers of AD

Chitinase-like protein 3 (Chi3l1), also known as YKL-40, is served as a key inflammatory biomarker predominantly expressed in both microglia and astrocytes of brain (Zeng et al. 2023). In CNS, Chi3l1/YKL-40 originates primarily from astrocytes and rare white matter neurons, rather than infiltrating macrophages (Craig-Schapiro et al. 2010; Querol-Vilaseca et al. 2017). Astrocytic Chi3l1/YKL-40 transcription is induced by interleukin-1β (IL-1β) and interleukin-6 (IL-6) released from macrophages *via* a signalling axis involving the signal transducer and activator of transcription 3 and reticuloendotheliosis viral oncogene-related B/p50 transcription complex (Bhardwaj et al. 2015). Despite of Chi3l1/YKL-40 well-established expression profile, the precise regulatory mechanisms of Chi3l1/YKL-40 in AD pathogenesis remain incompletely understood. Pathologically, elevated Chi3l1/YKL-40 expression correlates with cortical thinning, hippocampal atrophy and cognitive decline in AD. In Chi3l1/YKL-40 transgenic mouse models of AD, Chi3l1/YKL-40 is predominantly expressed in human astrocytes, Chi3l1/YKL-40-positive reactive astrocytes significantly increases during the late stages of AD (Zeng et al. 2023). The Chi3l1/YKL-40 upregulation is spatially associated with ionised calcium-binding adaptor molecule 1-positive microglia, suggesting a potential crosstalk between astrocytes and microglia in neuroinflammation. However, Chi3l1/YKL-40 in CSF concentrations do not reliably distinguish between typical AD phenotype (e.g., hippocampal sclerosis) and atypical AD variant phenotype (Baldacci et al. 2017). Clinically, Chi3l1/YKL-40 levels in CSF is correlated with both T-tau and P-tau in CSF and neuroimaging parameters such as cortical thickness and grey matter volume, which indicates Chi3l1/YKL-40 potential as a marker of neuroinflammation-linked neurodegeneration in AD. Notably, Chi3l1/YKL-40 has been shown to be correlated with both T-tau and neurofilaments like neurofilament light chain (NF-L) in non-amyloid CSF biomarker profiles (Hall et al. 2016; Melah et al. 2016). Collectively, Chi3l1/YKL-40 as emerges a promising CSF biomarker for astrocyte activation in AD and other dementia spectrum disorders (Baldacci et al. 2019), especially in the context of tau-related pathological cascades. However, the differential impacts of early versus late astrocyte activation on clinical disease progression remain unclear and warrant systematic investigation.

### The blood biomarkers of AD

Neurofilament pentraxins, a family of pre-synaptic proteins predominantly expressed in excitatory neurons, are aberrantly upregulated in the brains of patients with sporadic late-onset AD (Gómez De San José et al. 2022). This family includes neuronal pentraxin 1 and neuronal pentraxin 2 (NPTX2). Emerging evidences from cell and animal models demonstrate that NPTX2 interacts with complement protein C1q (C1q), preventing C1q activation and thereby inhibiting complement-mediated neuronal damage (Dejanovic et al. 2018; 2022; Gómez De San José et al. 2022). Notably, the adeno-associated virus-mediated overexpression of NPTX2 mitigates C1q-induced synapse loss and reduces microglia-driven neuronal destruction (Zhou et al. 2023). In inherited ADs, reduced NPTX2 levels are correlated with elevated C1q concentrations, leading to hyperactive complement signalling and synaptic imbalance-pathways that exacerbate ADs progression (Zhou et al. 2023). These findings highlight NPTX2 as a critical modulator of complement activity and microglia-mediated synaptopathy, offering new insights into ADs pathogenesis and potential diagnostic biomarkers (Gómez De San José et al. 2022).

**Figure 1.**
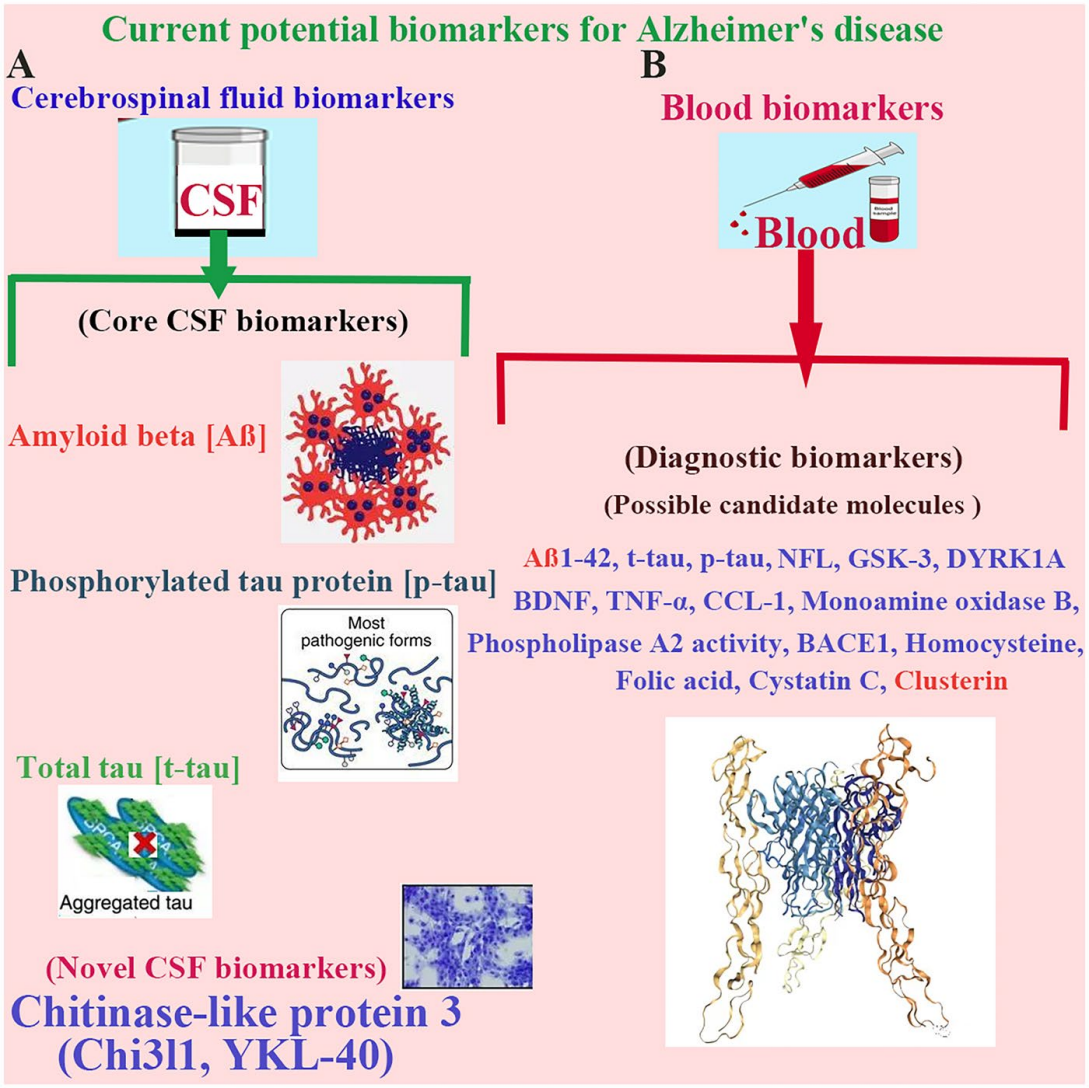
Current potential biomarkers for Alzheimer’s disease. (A) Current potential biomarkers for AD in CSF, such as, core CSF biomarkers Aβ, p-tau and t-tau, and novel CSF biomarkers Chi3l1 (KYL-40). **(B)** Current potential biomarkers for AD in blood, such as, the candidate molecules of diagnostic biomarkers. Abbreviation: AD, Alzheimer’s disease; Aβ, amyloid beta; BACE1, beta-site amyloid precursor protein cleaving enzyme 1; BDNF, Brain-derived neurotrophic factor; CCL-1, chemokine (C-C motif) ligand (CCL)-1; Chi3l1 (YKL-40), chitinase-like protein 3; CSF, cerebrospinal fluid; DYRK1A, Dual-specificity tyrosine phosphorylation-regulated kinase 1A; GSK-3, Glycogen synthase kinase 3; NFL, Neurofilament light chain; p-tau, phosphorylated tau protein; TNF-alpha (α), tumour necrosis factor-alpha (α); t-tau, total tau protein.

Being similar to classical AD pathogenic proteins, non-coding RNAs, particularly microRNAs (miRNAs) emerge as promising novel biomarkers for AD diagnosis (Wang and Zhang 2020). Exosomal miRNAs dysregulated in AD pathogenesis influence genes involved in APP processing, tau phosphorylation, mitochondrial function and apoptosis, enhancing their roles in orchestrating neurodegenerative cascades (Gaetani et al. 2020; Wang and Zhang 2020; Soares Martins et al. 2021). For example, Tau pathway regulation, miRNAs such as miR-92a-3p, miR-320a and miR-320b bind to MAPT mRNA, directly suppressing tau transcription and reducing brain tau levels (Long et al. 2025). Endogenous regulators of AD-related pathways miR-132-3p downregulation is linked to AD and tauopathies, miR-132-3p loss promotes tau aggregation, highlighting miR-132-3p protective role against tau misfolding (Smith et al. 2015). Multiple miRNAs such as miR-124 and miR-34a modulate tau phosphorylation by targeting kinases like GSK-3β, CDK5 and DAPK1, influencing tau toxicity (Smith et al. 2015). MiR-9, miR-29, miR-29a/b-1, miR-124, miR-101, miR-107, miR-298 and miR-328 are related to Aβ production increase. miR-149, miR-34a-5p, miR-16, miR-29c and miR-374b-5p are inversely correlated with beta-site amyloid precursor protein cleaving enzyme 1 (BACE1) mRNA expression to play anti-AD roles. miR-106a, miR-520c, miR-16, miR-17, miR-101 and miR-16 mediate the regulation of APP production. miR-132, miR-425-5p, miR-124-3p, miR-512, miR-125b-5p and miR-483–5p are linked to enhance or reduce tau phosphorylation. MiR-155, miR-146a, miR-146 and miR-132 are implicated in the regulation of inflammation. miR-484, miR-132 and miR-212 exert a significant effect on modulating synaptic function and neurotransmission. miR-107, miR-203 and miR-195 levels show a genetic correlated with APOE4 alleles and APOE E3/E3 genotype (Li et al. 2024). miR-15a and miR-185 inhibit GSK-3β expression, reduce APP phosphorylation and Aβ production in Aβ pathway regulation. miR-195 and miR-101 suppress CDK5, altering APP processing and Aβ deposition. The direct regulation of β-secretase 1 (BACE1) by miRNAs such as miR-29c and miR-128 modulates APP cleavage and Aβ load (Liu et al. 2014). Changes in miRNAs expression thus reflect both tau and Aβ pathological states, positioning them as dual-functional biomarkers for AD. Moreover, miRNAs ability to regulate multiple pathogenic nodes like synapse integrity and inflammation suggests potential as therapeutic targets. However, large-scale clinical validation across diverse cohorts and high-quality mechanistic studies are essential to establish their diagnostic and prognostic utility (Figure 1).

NF-L gene, a neuronal cytoplasmic protein being integral to myelinated axons, exhibits elevated plasma levels that correlate with Aβ deposition and tau protein pathology in AD. Nevertheless, NF-L utility as a specific biomarker for AD is constrained by minimal longitudinal changes in atrophy and potential modulation by cognitive decline and amyloid clearance mechanisms (Arslan et al. 2024). In biological matrices, NF-L levels in CSF and blood mirror the severity of axonal injury, presymptomatic elevations can be detected in years prior to clinical onset in familial AD mutation carriers (Monteiro et al. 2023). This temporal pattern underscores NF-L role as a sensitive indicator of neurodegeneration, further validated by strong correlations between plasma and CSF NF-L concentrations (Hansson et al. 2023). Similar with NF-L, plasma glial fibrillary acidic protein (GFAP) serves as a representative marker for reactive astrocytosis, displaying early upregulation during Aβ accumulation that precedes cognitive decline. Notably, plasma GFAP demonstrates a close correlation with cerebral Aβ burden compared to GFAP CSF counterpart, and a distinction attributed to latter compromised stability (Pereira et al. 2021). While GFAP lacks a disease specificity, its dynamic range is significantly broader in AD than in non-AD neurodegenerative disorders such as frontotemporal dementia, highlighting its potential as a discriminative biomarker in differential diagnosis (Arslan et al. 2024).

### The imaging biomarkers of AD

Magnetic resonance imaging (MRI) provides the direct visualisation of brain atrophy. Quantitative structural MRI studies demonstrate atrophy in the multiple brain regions of AD patients. Additionally, quantitative structural MRI technique monitors changes in cerebral hemodynamics to reflect neural activity, enabling the evaluation of activation patterns and functional connectivity in different brain regions. Magnetic resonance spectroscopy measures neurochemical substances, the concentration or ratio of metabolic products of neurochemical substances can be served as diagnostic biomarkers for AD (Dang et al. 2023). Positron emission tomography (PET) uses radioactive tracers to assess metabolic changes, plays a pivotal role in AD diagnosis and the identification of prognosis. However, PET usage is limited by high costs and radiation exposure. Major technologies of PET include fluorodeoxyglucose, amyloid and Tau PET. Fluorodeoxyglucose PET can detect glucose metabolism alterations, reduced glucose metabolic activity is served as the biomarker of neurodegeneration in AD. Amyloid and Tau PET can visualise amyloid plaques and tau protein deposition respectively, facilitating AD diagnosis and staging.

Single-photon emission computed tomography (SPECT) can detect cerebral blood flow and metabolic changes, offering practical value in AD diagnosis despite of lower spatial resolution compared to PET and MRI. Optical imaging technologies can visualise molecular-level changes in AD brain but face significant challenges in clinical implementation, such as signal interference and limited tissue penetration depth (Dang et al. 2023). AD pathology extends beyond CNS to affect visual system. Retinal nerve fibre layer (RNFL) and optic nerve maintain direct connections with brain, harbour AD-related molecular signatures (Femminella et al. 2018). The pathological changes of visual system in AD patients include the apoptosis of retinal ganglion cells, RNFL thinning and the structural abnormalities of optic nerve. Retinal imaging modalities like optical coherence tomography and optical coherence tomography angiography have gained a widespread use in AD diagnosis (Prasath and Sumathi 2023). Retinal imaging finds increased thickness in retinal and choroidal layers, juxtaposed with reduced RNFL and overall retinal thickness in AD patients. Advanced retinal image processing techniques including reconstruction, filtering, segmentation and registration allow to detailed analyse retinal structural and functional changes, being helpful to discover specific AD biomarkers associated with retinal damage (Casaletto et al. 2017). Neuroimaging methods exhibit not only distinct advantages but also limitations in detecting AD biomarkers, often providing complementary evidences for AD diagnosis. Factors such as age, sex and educational level influence AD imaging outcomes, necessitating their explicit integration into the clinical data interpretation of neuroimaging.

### The emerging biomarkers of AD

Diagnostic landscape for AD is undergoing a paradigm shift with the rise of non-invasive biomarkers in biofluids such as saliva, urine and tears. These emerging markers and detected technologies offer unique advantages but also carry inherent limitations. Though standardisation and longitudinal validation remain critical for clinical translation, the combined usage of multiple biomarker approaches and technical integration are increasingly utilised to enhance diagnostic accuracy.

While PET-based biomarkers and CSF biomarkers have the revolutionised change of AD diagnosis, they are not without flaws, such as PET involves radiation exposure and high costs, while CSF sampling is invasive. In this context, blood-based biomarkers have emerged as a promising alternative. Concurrently, saliva represents the valuable non-invasive source of biomarkers, with lactoferrin showing reduced concentrations of saliva in AD patients and mild cognitive impairment patients, making it a candidate biomarker for early detection of AD. Salivary acetylcholinesterase activity is also diminished in AD patients, but correlating with therapeutic response to cholinesterase inhibitors (Ashton et al. 2019).

Tears hold an equal promise of biomarker because T-tau and Aβ42 proteins and microRNAs can be detected in tears, are emerged as potential biomarkers. The advantages of tear-based assays like easy non-invasive sampling and the repeatable make tears to be attractive tools for longitudinal monitoring of AD (Kaštelan et al. 2023).

Retina has emerged as a window of AD pathology because of Aβ deposition, RNFL thinning and vascular parameter alterations in retina, and demonstrating strong associations with cerebral AD biomarkers (Majeed et al. 2021). Similarly, urine harbours biomarker candidates like 8-hydroxy-2’-deoxyguanosine (8-OHdG, a marker of oxidative stress) and neuron-specific enolase subtype AD7c-NTP, but their clinical utility requires further validation (Villa et al. 2020).

## Parkinson’s disease

### The pathophysiologies of PD

PD is a chronic neurodegenerative disorder characterised by the progressive loss of dopaminergic neurons in substantia nigra. As disease advances, both motor symptoms including tremor, rigidity, bradykinesia and postural balance dysfunction and non-motor symptoms such as olfactory impairment, sleep disorders, autonomic nervous system dysfunction and neuropsychiatric abnormalities gradually appear. While motor symptoms are closely linked to dopamine depletion in substantia nigra, non-motor symptoms often precede motor onset by 5–10 years and occur independently of dopamine loss (Sechi and Sechi 2024). The traditional diagnosis of PD mainly relies on clinical symptoms, which hinders the detection of early pathophysiological changes. Therefore, there is an urgent need for reliable biomarkers to enable early PD diagnosis and the accurate prognosis assessment of PD progression (Ciechanover and Kwon 2015).

A defining pathological hallmark of PD is aggregated α-synuclein, which forms lewy bodies within neurons and neurites across both central and peripheral nervous systems. α-synuclein is predominantly expressed in presynaptic nerve terminals, where it regulates synaptic vesicle releasing and recycling. Similar to tau pathology in other NDs, α-synuclein exhibits self-propagating behaviour through template misfolding and cell-to-cell transmission (Sechi and Sechi 2024). The Lewy bodies formation resulted by α-synuclein deposition and accumulation in neuronal components and neurites and the subsequent induction of neuronal death are central to PD pathophysiology.

### The biomarkers of PD in CSF and blood

CSF and blood-based biomarkers, including α-synuclein, lysosomal enzymes, Aβ peptides, tau and NF-L, hold diagnostic and prognostic values for PD. Compared to healthy controls (HCs), PD patients exhibit lower CSF total α-synuclein levels but elevated oligomeric α-synuclein (o-αSyn) and phosphorylated α-synuclein (p-αSyn) (Xu et al. 2021). Elevated o-αSyn levels are also detected in the serum and red blood cells of PD patients. Reduced lysosomal enzyme activity in CSF further reflects PD-related neurodegeneration. While classical AD biomarkers such as CSF Aβ42, T-tau and P-tau are not only diagnostic for PD, but also aid in prognostic assessment. NF-L, the universal marker of axonal injury in NDs such as AD and ALS, is not specific to PD but NF-L in plasma correlates with the disease severity and progression of PD (Xu et al. 2021).

DJ-1, encoded by PARK7 gene, also known as PD protein 7, is causally linked to early-onset PD *via* loss-of-function (LOF) mutations (Lockhart et al. 2004). DJ-1 mutations underlie rare autosomal recessive PD cases, typically presenting with young-onset symptoms. DJ-1 multifunctional roles in PD include chaperone activity, transcriptional regulation, the modulation of protein degradation pathways and antioxidant function (Morrone Parfitt et al. 2024). A key pathological feature of PD is α-synuclein accumulation in midbrain, where DJ-1 deglycosylase activity protects DNA, proteins and lipids from harmful glycosylation. Oxidative damage in PD impairs proteome stability and cell viability, driving the accumulation of advanced glycation end products-critical drivers of neurodegeneration (Jun and Kool 2020). Oxidative stress is suggested to exert a key effect on PD, but clinical investigated results were inconsistent. A systematic review and meta-analysis demonstrated the higher blood concentrations of 8-hydroxy-2’-deoxyguanosine, malondialdehyde, nitrite and ferritin, while the lower blood concentrations of catalase, uric acid, glutathione and total-cholesterol in PD patients. This study strengthens clinical evidences that PD is accompanied by an oxidative stress increase (Wei et al. 2018).

In a DJ-1-deficient human midbrain organoid (hMIDO) model, lysosomal proteolysis is impaired, leading to advanced glycation end products (AGEs) accumulation, increased α-synuclein phosphorylation and protein aggregation. Astrocytes play a central role in these biological processes. DJ-1 loss diminishes their metabolic support capacity and induces a pro-inflammatory phenotype. DJ-1 expressing astrocytes rescue proteolytic deficits in DJ-1 knockout midbrain neurons, highlighting astrocytic dysfunction in DJ-1 LOF-mediated neurodegeneration (Morrone Parfitt et al. 2024). In hMIDO model, DJ-1 knockout triggers elevated methylglyoxal-derived hydroimidazolone (an early AGE modification), confirming enhanced glycosylation damage (Hasim et al. 2014; Choi et al. 2019). As brain primary glycolytic cells, astrocytes are particularly vulnerable to glycation stress (Tong et al. 2015). Astrocytes are pivotal for maintaining neuronal homeostasis and degrading damaged lipids and proteins derived from neurons. DJ-1 exerts significant neuroprotective effects on astrocytes, and PD-associated LOF mutations in DJ-1 drive neurodegeneration through astrocyte-mediated mechanism (di Domenico et al. 2019; Tsunemi et al. 2020; Streubel-Gallasch et al. 2021). Proteomic instability in astrocytes triggers inflammatory reactivation and cytokine release (di Domenico et al. 2019; Wilson et al. 2019; Streubel-Gallasch et al. 2021). In DJ-1 knockout hMIDO model, astrocyte proliferation is markedly increased, accompanying by elevated monomeric phosphorylated α-synuclein expression. It suggests that DJ-1 LOF promotes α-synuclein accumulation *via* astrocyte activation, linking inflammatory reactivation and cytokine release to pathological α-synuclein dynamics. Collectively, the current findings highlight DJ-1 in the CSF and blood-based as a potential biomarker for PD diagnostic utility.

**Figure 2.**
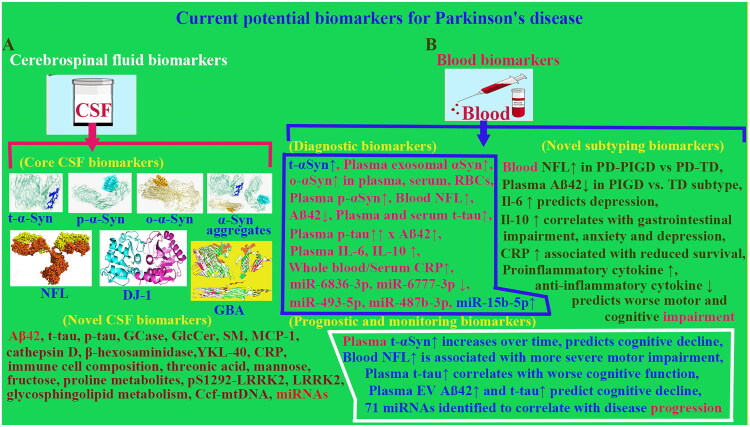
**Current potential biomarkers for Parkinson’s disease**. **(A)** Current potential biomarkers for PD in CSF, such as, core CSF biomarkers t-α-Syn, p-α-Syn, o-α-Syn, α-Syn aggregates, NFL, DJ-1 and GBA, and novel CSF biomarkers. **(B)** Current potential biomarkers for PD in blood, such as, diagnostic biomarkers, progression and prognostic monitoring biomarkers and novel biomarkers. Abbreviation: Aβ-42, amyloid beta; αSyn (α-Syn), α-Synnuclein; Ccf-mtDNA, circulating cell-free mitochondrial DNA; CRP, C-reactive protein; EV, extracellular vesicle; GBA, glucocerebrosidase; GCase, Glucocerebrosidase; GLcCer, Glucosylceramides; IL-1,6,10, interleukin-1,6,10; LRRK2, Leucine-rich repeat kinase 2; MCP-1, monocyte chemoattractant protein-1; miRNAs (miR), microRNAs; NFL, Neurofilament light chain; o-alpha(α)-Syn (o-αSyn), oligomeric-alpha(α)-synuclein; p-alpha(α)-Syn (p-αSyn), phosphorylated alpha(α)-synuclein protein; PD, Parkinson’s disease; PD-PIGD, Parkinson’s disease patients with postural instability and gait disorders; pS1292-LRRK2, total and phosphorylated (pS1292) LRRK2; p-tau, phosphorylated tau protein; RBCs, red blood cells; SM, sphingomyelin; t-alpha(α)-Syn (t-αSyn), TD, tremor-dominant PD; total alpha(α)-synuclein; t-tau, total tau protein; YKL-40 (Chi3l1), chitinase-like protein 3), chitinase-like protein 3.

Mutations in glucocerebrosidase (GBA) gene are the most common genetic risk factor for PD, which links to lysosomal dysfunction in sphingolipid metabolism. PD patients exhibit reduced GBA and cathepsin D activity independent of GBA mutation status, with diagnostic sensitivities/specificities of 67%/77% GBA and 61%/77% cathepsin D, respectively (Zhang et al. 2024). Combining GBA activity, o-αSyn and p-αSyn ratio and age achieves 82% sensitivity/71% specificity for early PD diagnosis. These data establish GBA as a robust diagnostic biomarker for PD (Figure 2).

### The imaging biomarkers of PD

Imaging biomarkers are playing an increasingly significant role in monitoring the progression of clinical trials and optimising clinical care management for PD. Clearly defining the diagnostic efficacy of various imaging biomarkers and their associative temporal evolution across different disease stages of PD holds immeasurable value for the clinical diagnosis of PD.

Dopaminergic positron emission tomography/single-photon emission computed tomography (PET/SPECT) imaging can detect dysfunction in dopaminergic system during the preclinical and prodromal stages of PD before clinical symptoms appear. While these examined results show a lack of significant correlation with actual clinical functional changes in PD patients yet. At the early stage of PD, dopaminergic PET/SPECT can effectively identify the loss of dopaminergic innervation, but sensitivity in monitoring PD progression decreases significantly two years after diagnosis. When PD progresses to mid-late stage, although dopaminergic PET/SPECT remains an effective biomarker for reflecting disease status, its ability to track the dynamic progression of PD has still certain limitations (Biondetti et al. 2020).

Non-dopaminergic PET imaging technologies, including the detection of serotoninergic and cholinergic systems as well as neuroinflammation-related markers, exhibit a certain degree of sensitivity to PD status, but the application potential of these imaging technologies as early diagnostic biomarkers for PD still needs further clinical validation. At the early stage of PD, intrinsic relationships between the change characteristics of serotoninergic and cholinergic biomarkers and disease severity or duration are not fully understood. However, at the mid-late stage of PD, changes in serotoninergic and cholinergic neural activity show a clear correlation with cognitive decline and the exacerbation of clinical symptoms in PD patients (Van Der Zee et al. 2021).

Diffusion MRI imaging technology enables the quantitative assessment of early neurodegenerative changes during the preclinical and prodromal stages of PD, for example, an increase in free water content in substantia nigra region can be observed in PD patients with idiopathic rapid eye movement sleep behaviour disorder (iRBD). At the early stage of PD, an increased free water content in posterior substantia nigra makes it become a promising biomarker for reflecting disease status and progression. At the mid-late stage of PD, an increased free water in anterior substantia nigra can serve as an effective indicator for monitoring disease progression (Gaurav et al. 2021).

Neuromelanin-sensitive MRI can observe a reduction in neuromelanin signal intensity in substantia nigra region during the preclinical and prodromal stages of PD, suggesting its potential application value in assessing prodromal disease status. At the early stage of PD, this technology is a reliable early diagnostic biomarker and can also be used to track disease progression. As disease progresses to mid-late stage, neuromelanin signal further attenuates, continuing to play an important role in monitoring disease progression (Gaurav et al. 2021).

Iron-sensitive MRI imaging technology shows certain application prospects during the preclinical and prodromal stages of PD, but the effectiveness of its use as a biomarker for prodromal disease status still requires more research for validation. At the early stage of PD, this technology can serve as a reliable basis for judging disease status, but the evidence for tracking disease progression is insufficient. When PD enters mid-late stage, it still remains a reliable biomarker for assessing disease status, among which R2* parameter may have higher sensitivity to PD progression (Mitchell et al. 2021).

### The emerging biomarkers of PD

iRBD is recognised as a prodromal marker of synucleinopathy, while speech serves as a sensitive indicator of motor function. Dr. Jan Rusz conducted a multicentre study involving patients with iRBD and PD. The study findings reveal that automatic speech analysis may serve as a biomarker for evaluating PD progression and the efficacy of therapeutic interventions. Although phonetic differences exist across languages, overall trends in relationship between automatic speech analysis and disease assessment are consistent (Rusz et al. 2021).

## Amyotrophic lateral sclerosis

### The pathophysiologies of ALS

ALS is a rare adult-onset neurodegenerative disease, pathophysiologically characterised by selective degeneration of upper and lower motor neurons in cerebral motor cortex, brainstem motor nuclei and spinal cord anterior horn, which leads to progressive muscle weakness and atrophy, ultimately respiratory failure caused by serious respiratory muscle atrophy and weakness, leading to death. Clinically, ALS presents with a combination of classic lower motor neuron and upper motor neuron signs, including muscle atrophy, fasciculations, altered muscular tension (hypotonia or spasticity), hyperreflexia and pathological reflexes. ALS currently has not any effective measure curing and preventing it progression yet, the average lifetime of ALS is around 3–5 years after diagnosis (Darabi et al. 2024). Globally, an estimated 223,000 survival individuals live with ALS, a number projected to increase by 69% by 2040 due to ageing populations worldwide. This growing burden underscores the urgency of improving our understanding of ALS pathogenesis and developing effective interventions (Vejux et al. 2018). Therefore, developing the accurate and confident diagnostic biomarker is very necessary and urgent for early diagnosis of ALS in order to early intervene ALS pathophysiologies.

Clinically, ALS is commonly classified into two major forms of familial ALS (fALS) and sporadic ALS (sALS). Among them, fALS accounts for 5–10% of cases, characterised by Mendelian inheritance patterns, sALS comprises about 90% of cases, no presently found clear genetic aetiology (Morimoto et al. 2019). Although ALS is influenced by complex gene-environment interactions, accumulating evidences highlight that ALS risk arises from complex interactions between genetic variations and environmental factors (Mathis et al. 2019). Like other NDs, ALS is also characterised pathophysiologically by misfolded protein accumulation, among them, the most notably is TAR DNA binding protein 43 kDa (TDP-43), it undergoes pathological modifications such as phosphorylation and mislocalization to produce toxic protein form to damage neuron in the pathophysiology of ALS (Suk and Rousseaux 2020). In addition, neurodegenerative pathophysiologies in ALS is further accompanied by neuroinflammation, microgliosis and astrogliosis activation (DeJesus-Hernandez et al. 2011). While the exact pathogenesis in ALS remains incompletely understood, current researches implicate mechanisms including mitochondrial dysfunction, mutational effects in superoxide dismutase 1 (SOD1), TDP-43 and other pathogenic genes, and glutamate excitotoxicity (Bonafede and Mariotti 2017; Yuan et al. 2021). Among them, genetic factors are widely recognised by scientists from the 1993 discovery of SOD1 mutations in fALS and identifying subsequently ALS-linked genes such as TDP-43, involved in RNA processing and protein synthesis (Vejux et al. 2018; Mathis et al. 2019; Morimoto et al. 2019; Darabi et al. 2024). Therefore, pathogenic proteins from these genes mutations accumulate in extracellular vesicles derived from plasma and CNS, positioning them as promising diagnostic biomarkers for ALS (Ferrara et al. 2018; Silverman et al. 2019).

### The genetic biomarkers of ALS in CSF and blood

TDP-43 is a central biofluid biomarker for ALS, with cytoplasmic deposition observed in approximately 97% of ALS patients across affected tissues (Sreedharan et al. 2008; Hayes and Kalab 2022). The RNA-binding protein encoded by TDP-43 gene is critical for RNA processing, splicing and stabilising (Majumder et al. 2018; Prasad et al. 2019). TDP-43 normally localises in nucleus, while shuttles dynamically from nucleus to cytoplasm at pathological condition, where it assembles into RNA transport granules in neuronal cell bodies and dendrites. These granules are essential for local protein synthesis in dendrites, a vital process for neuronal plasticity (Suk and Rousseaux 2020). In nucleus, TDP-43 regulates the microRNA biogenesis and splicing of hundreds of pre-mRNAs, including its own transcript, thereby maintaining homeostatic protein levels through auto-regulatory feedback. Cytoplasmic TDP-43 contributes to axonal transport and stress granule formation, ensuring neuronal resilience to cellular stress (Suk and Rousseaux 2020). In ALS, TDP-43 undergoes pathological post-translational modifications, such as ubiquitination, hyperphosphorylation and C-terminal truncation. These changes of TDP-43 enhance its aggregation propensity, leading to widespread neurotoxicity and motor neuron death. Dysregulated auto-regulation of TDP-43 mRNA stability further exacerbates cytoplasmic mislocalization, creating a vicious cycle of protein aggregation and neurodegeneration (Suk and Rousseaux 2020). Global TDP-43 knockout in mice causes embryonic lethality, underscoring TDP-43 essential role in early development (Prasad et al. 2019). However, conditional motor neuron-specific TDP-43 depletion induces progressive motor deficits and TDP-43 proteinopathy, mirroring ALS pathology (Majumder et al. 2018). Selective overexpression or knockdown of TDP-43 in glial cells or muscle tissue also recapitulates ALS-like phenotypes (Prasad et al. 2019), highlighting cell-nonautonomous mechanisms in the disease progression of ALS. Transgenic models expressing mutant TDP-43 demonstrate conformational changes (misfolding) that disrupts both loss- and gain-of-function pathways. Pathologically, TDP-43 related ALS is characterised by a hallmark of TDP-43 mislocalization, such as granular, bunched or large cytoplasmic TDP-43 aggregates accompanied by nuclear depletion (Suk and Rousseaux 2020). Understanding TDP-43 dual roles in normal physiology and pathophysiology is critical for developing the biomaker of targeting diagnostics and therapies. Protein presence in biofluids like CSF and plasma, and its association with disease severity make TDP-43 a validated biomarker for ALS.

Additionally, the most prevalent genetic abnormality in fALS is hexanucleotide repeat expansion in the intronic region of chromosome 9 open reading frame 7 (C9orf72) gene. The longitudinal studies of C9orf72 hexanucleotide repeat expansion carriers have illuminated mechanisms underlying early TDP-43 aggregation, offering critical insights into pre-symptomatic prevention, intervention refinement and diagnostic biomarkers for ALS spectrum disorders (Mizielinska et al. 2025). These findings not only enhance our ability to pre-diagnose ALS but also provide valuable clues for identifying novel diagnostic biomarkers by linking genetic perturbations to early pathological cascades.

SOD1 is a highly abundant cytoplasmic and mitochondrial enzyme, it neutralises their toxic by catalysing superoxide radicals to decompose into molecular oxygen and hydrogen peroxide (Huai and Zhang 2019). As one of the earliest identified ALS-associated proteins, SOD1 was the first to be detected in extracellular vesicles (EVs), alongside TDP-43 and C9orf72 (Vu and Bowser 2017). Misfolded, disulfide-crosslinked SOD1 aggregates are observed in EVs derived from spinal motor neurons and glial cells in most familial fALS cases and a subset of sALS cases (Fetherolf et al. 2017). Notably, while SOD1 mutations are rare in fALS and virtually absent in sALS, wild-type SOD1 overexpression is a common feature across both subtypes of ALS (Tiwari et al. 2005). *In vitro* studies demonstrate that wild-type SOD1 can self-propagate, aggregate and transfer between cells, mirroring prion-like propagate manner. Similarly, SOD1 mutant aggregating in EVs exhibits the prion-like propagate manner in ALS pathology, analogous to proteinopathies driven by misfolded protein deposition in AD and PD. Despite precise molecular mechanisms linking SOD1 mutations to neurotoxicity remain incompletely understood, based on above insights, SOD1 served as a candidate genetic biomarker still is recognised.

As above described studies, one of the most common genetic abnormalities in ALS is hexanucleotide repeat expansion mutation in C9orf72, which accounts for approximately 40% of fALS cases (Tiwari et al. 2005; Sreedharan et al. 2008; DeJesus-Hernandez et al. 2011; Renton et al. 2011; Bonafede and Mariotti 2017; Fetherolf et al. 2017; Vu and Bowser 2017; Ferrara et al. 2018; Majumder et al. 2018; Huai and Zhang 2019; Prasad et al. 2019; Silverman et al. 2019; Yuan et al. 2021; Hayes and Kalab 2022; Mizielinska et al. 2025). This mutation induces toxic gain-of-function effects in C9orf72, concurrently disrupting its normal cellular roles (Mathis et al. 2019). Both SOD1 and C9orf72 mutations are associated with dysregulated immune responses, autophagic dysfunction and glial cell abnormalities, which also occurs in ALS patients without these genetic alterations. Notably, anti-inflammatory pathways predominate during the early stages of ALS, whereas pro-inflammatory mechanisms become dominant at later stages, accelerating motor neuron degeneration. These findings suggest that inflammation-related biomarkers could provide critical insights into disease staging, progression rates, pathophysiological mechanisms and potential therapeutic pathways in ALS.

miRNAs is small non-coding RNA molecules, acts as the endogenous regulators of gene expression, have emerged as promising biomarkers of NDs such as AD, PD and ALS, because their dysregulated expression is detectable in human biofluids (Rajgor 2018). Among them, miR-206 has garnered significant attention due to its elevated levels in ALS patients, though its circulating concentrations decline as disease progresses (Freischmidt et al. 2015; Waller et al. 2017; Ravnik-Glavač and Glavač 2020). Other miRNAs like miR-338-3p and miR-143-3p show altered expression in both CSF and serum of ALS patients, is potential biomarker candidates of ALS. Moreover, motor neuron-enriched miR-218 increases in the CSF of ALS rodent models, correlates with the number of surviving spinal motor neurons, holds potential as a biomarker for assessing motor neuron amount and disease progression (Waller et al. 2017; Dobrowolny et al. 2021). Mechanistically, miR-338-3p is implicated in multiple pathways driving ALS pathogenesis, including neurodegeneration and apoptosis. The upregulation of miR-338 and miR-181a-5p contributes to cell death *via* apoptotic mechanisms. The combinatorial application of biomarker approaches, such as ratios of miR-181a-5p/miR-15b-5p or miR-181a-5p/miR-21-5p, enhance diagnostic specificity compared to the usage of single miRNAs biomarker (Ricci et al. 2018). Circulating miRNAs involving in neurodegeneration like miR-338, miR-142, miR-183 and let-7d as well as muscle physiology like miR-206, miR-133a, miR-133b and miR-27a also participate in ALS pathophysiological processes. Muscle-specific miRNAs like miR-206, miR-133a and miR-133b regulate muscle proliferation and regeneration, and show consistent upregulation in ALS patients. miR-206 specifically promoting neuromuscular junction reinnervation after nerve injury, serving as a validated circulating biomarker (Ricci et al. 2018). Differential miRNA expression analyses suggest that miR-183 may be specific biomarker to sALS, while miR-451 and miR-3935 act as more general NDs biomarkers (Ricci et al. 2018). Despite of their utility, miRNA changes are not exclusive to ALS and may overlap with other NDs or ALS-like disorders, their specificity and utility necessitating further validation.

Beyond miRNAs, long non-coding RNAs and circular RNAs (Ravnik-Glavač and Glavač 2020), being proposed for helping to diagnosis some NDs (Lee and Woodruff 2021). For example, the elevated levels of circular RNA hsa_circ_0023919 have been observed in blood samples (Dejanovic et al. 2022) and CSF from sALS patients (Lee and Woodruff 2021), which further indicates potential uses for the other types of RNA in diagnosing ALS. Combinatorial multiple different types of RNA biomarkers and combining RNA biomarkers from serum and CSF demonstrate higher diagnostic accuracy than the use of single RNA biomarkers, which addresses the limitations of individual biomarker specificity, and offering promise for improving the biomarkers used effects of ALS diagnosis.

### The neurofilaments biomarkers of ALS

Neurofilaments represent the most extensively studied biomarkers for ALS (Bacioglu et al. 2016). As intermediate filaments, neurofilaments consist of four subunits, heavy chain (NF-H), medium chain and NF-L neurofilaments, along with α-internexin in CNS (Miteva et al. 2023). These neurofilaments undergo diverse post-translational modifications, among them, phosphorylation is the most critical. Substantial evidences support neurofilament levels as diagnostic biomarkers for ALS (Ishigaki and Sobue 2018). At present, multiple studies demonstrate that the levels of NF-L and phosphorylated NF-H (pNF-H) are significantly elevated in the CSF of ALS patients compared to HCs, and detect diagnostic sensitivity and specificity for ALS to exceed 80% (Hayes and Kalab 2022). Moreover, the fluctuations of neurofilaments biomarkers levels are correlated with the disease progression and survival rates of ALS patients. Notably, regardless of patient age, NF-L levels in CSF are detected to be strongly associated with the disease progression of ALS, higher NF-L concentrations are consisted with the increased upper motor neuron damage or more rapid disease progression of ALS (Lu et al. 2015; Gendron et al. 2017; Poesen and Van Damme 2018). NF-L levels in both serum and CSF exhibit a high degree of correlation with ALS (Lu et al. 2015; Gendron et al. 2017; Poesen and Van Damme 2018). Compared to HCs, ALS patients show significantly elevated blood NF-L levels, and baseline NF-L levels serve as a robust independent predictor of survival time, the higher initial levels of NF-L are negatively correlated with shorter survival time (Brettschneider et al. 2006). Importantly, NF-L levels do not change significantly as the disease progresses of ALS, thus, positioning NF-L as biomarkers for ALS diagnosis and prognosis rather than for monitoring the biomarker of ALS progression (Brettschneider et al. 2006; Benatar et al. 2020). Both NF-L and pNF-H act as the biomarkers of axonal damage, directly reflecting neuronal health. However, NF-L demonstrates limited specificity for ALS diagnosis (Boylan et al. 2013; Ganesalingam et al. 2013). In contrast, CSF pNF-H emerges as a superior biomarker for differentiating ALS from other NDs and similar disorders.

### The inflammation biomarkers of ALS

Inflammatory factors also represent potential biomarkers for ALS. Multiple studies have demonstrated the significantly elevated blood levels of inflammatory factors such as tumour necrosis factor-α (TNF-α), IL-1β, IL-6, interleukin-8 (IL-8), and vascular endothelial growth factor in ALS patients, which suggests that TNF-α, IL-1β, IL-6 and IL-8 are the potential candidates of biomarkers in ALS (Lockhart et al. 2004; Tiwari et al. 2005; Brettschneider et al. 2006; Sreedharan et al. 2008; DeJesus-Hernandez et al. 2011; Renton et al. 2011; Hasim et al. 2014; Liu et al. 2014; Ciechanover and Kwon 2015; Freischmidt et al. 2015; Lu et al. 2015; Smith et al. 2015; Tong et al. 2015; Bacioglu et al. 2016; Bonafede and Mariotti 2017; Casaletto et al. 2017; Fetherolf et al. 2017; Gendron et al. 2017; Vu and Bowser 2017; Waller et al. 2017; Femminella et al. 2018; Ferrara et al. 2018; Majumder et al. 2018; Poesen and Van Damme 2018; Rajgor 2018; Ricci et al. 2018; Vejux et al. 2018; Wei et al. 2018; Ashton et al. 2019; Choi et al. 2019; di Domenico et al. 2019; Huai and Zhang 2019; Mathis et al. 2019; Morimoto et al. 2019; Prasad et al. 2019; Silverman et al. 2019; Wilson et al. 2019; Biondetti et al. 2020; Gaetani et al. 2020; Jun and Kool 2020; Ravnik-Glavač and Glavač 2020; Suk and Rousseaux 2020; Tsunemi et al. 2020; Villa et al. 2020; Dobrowolny et al. 2021; Gaurav et al. 2021; Lee and Woodruff 2021; Majeed et al. 2021; Mitchell et al. 2021; Pereira et al. 2021; Rusz et al. 2021; Streubel-Gallasch et al. 2021; Van Der Zee et al. 2021; Yuan et al. 2021; Hayes and Kalab 2022; Dang et al. 2023; Hansson et al. 2023; Kaštelan et al. 2023; Miteva et al. 2023; Monteiro et al. 2023; Prasath and Sumathi 2023; Arslan et al. 2024; Darabi et al. 2024; Li et al. 2024; Morrone Parfitt et al. 2024; Sechi and Sechi 2024; Zhang et al. 2024; Long et al. 2025; Mizielinska et al. 2025) Several investigations have observed alterations in blood immune cell profiles including granulocytes and T cells, which implies that the profiles alteration of blood immune cells also may be the potential candidates of ALS diagnostic biomarkers. Meanwhile, it identifies phenotypic changes in blood including IL-6, IL-8, TNF and interferons yet, which further suggests inflammatory factors such as IL-6, IL-8, TNF and interferons be potential candidate biomarkers for ALS (Ganesalingam et al. 2013; Brodovitch et al. 2021). Collectively, current evidences indicate that distinct immune regulatory patterns are produced in ALS compared to HCs, which is confirmed by multiple different methods of studies. Among them, transcriptional-level studies have provided novel insights, revealing a pro-inflammatory gene signature characterised by upregulated IL-8 and FBJ murine osteosarcoma viral oncogene homolog B in ALS patients. Moreover, macrophages produce response to interferon-γ from activated T lymphocytes in ALS patients. These findings have led to the identification of neopterin as an additional inflammatory biomarker for ALS (Shepheard et al. 2022). neopterin serves as a systemic marker of immune activation and is renally excreted, thus it can easily detected noninvasively urinary neopterin, which urinary neopterin become a conveniently and noninvasively detected biomarker. The results of urinary neopterin detection showed that the elevated urinary neopterin levels of ALS patients are correlated with severer clinical symptoms measured by revised amyotrophic lateral sclerosis functional rating scale (ALSFRS-R), which indicates urinary neopterin is a promising biomarker identifying the severity of ALS (De Paula Martins et al. 2018).

### Other biomarkers of ALS

Cystatin C, an endogenous cysteine protease inhibitor, protects motor neurons against damage by promoting autophagy and inhibiting cathepsin B-mediated neurotoxicity (Watanabe et al. 2014). In addition, cystatin C plays a critical role in regulating extracellular protein homeostasis within CNS (Zhu et al. 2018). Theoretically, cystatin C is a candidate of ALS biomarker.

Transthyretin (TTR) is a highly conserved homo-tetrameric protein primarily synthesised in choroid plexus epithelial cells and hepatocytes, with secretion into CSF and plasma, respectively. TTR is also expressed in neurons, where it influences neurogenesis, nerve repair and axon growth. Multiple studies demonstrate the progressive decline of TTR in ALS accompanies with reduced TTR levels in spinal cord, which likely attributes to motor neuron loss and diminishes TTR expression in surviving motor neurons. Therefore, TTR alternation in CSF and plasma may be a candidate biomarker for ALS.

C-reactive protein (CRP) an inflammatory acute-phase protein regulated by pro-inflammatory cytokines, is secreted by hepatocytes during inflammatory responses. Current evidence shows that ALS patients with higher serum CRP levels exhibit more rapid disease progression compared to those with lower CRP levels (Darabi et al. 2024). Additionally, serum CRP levels correlate significantly and negatively with ALSFRS-R scores (Darabi et al. 2024), indicating their utility as feasible prognostic markers in ALS.

The CSF levels of basic fibroblast growth factor are elevated in ALS and correlate with disease duration and survival time. Similarly, the plasma levels of perivascular fibroblast-secreted phosphoprotein-1 (SPP-1, also known as osteopontin) are increased in ALS patients. Elevated SPP-1 levels at diagnosis ALS predict shorter survival (Vidovic et al. 2023). Thus, bFGF and SPP-1 levels are the candidates of prognostic estimated biomarkers.

Serum uric (UA) acid levels inversely correlate with the mortality risk of ALS, ALS patients exhibiting lower UA levels than HCs. UA decline correlates with ALS progression, supporting a role for oxidative stress in ALS initiation and spread (Lockhart et al. 2004; Tiwari et al. 2005; Brettschneider et al. 2006; Sreedharan et al. 2008; Keizman et al. 2009; Craig-Schapiro et al. 2010; DeJesus-Hernandez et al. 2011; Renton et al. 2011; Boylan et al. 2013; Ganesalingam et al. 2013; Hasim et al. 2014; Liu et al. 2014; Watanabe et al. 2014; Bhardwaj et al. 2015; Ciechanover and Kwon 2015; Freischmidt et al. 2015; Lu et al. 2015; Smith et al. 2015; Tong et al. 2015; Bacioglu et al. 2016; Hall et al. 2016; Melah et al. 2016; Baldacci et al. 2017; Bonafede and Mariotti 2017; Casaletto et al. 2017; Fetherolf et al. 2017; Gendron et al. 2017; Querol-Vilaseca et al. 2017; Vu and Bowser 2017; Waller et al. 2017; De Paula Martins et al. 2018; Dejanovic et al. 2018; Femminella et al. 2018; Ferrara et al. 2018; Ishigaki and Sobue 2018; Majumder et al. 2018; Poesen and Van Damme 2018; Rajgor 2018; Ricci et al. 2018; Vejux et al. 2018; Wei et al. 2018; Zhu et al. 2018; Ashton et al. 2019; Baldacci et al. 2019; Choi et al. 2019; di Domenico et al. 2019; Huai and Zhang 2019; Mathis et al. 2019; Morimoto et al. 2019; Prasad et al. 2019; Rösler et al. 2019; Silverman et al. 2019; Wilson et al. 2019; Benatar et al. 2020; Biondetti et al. 2020; Gaetani et al. 2020; Jun and Kool 2020; Ravnik-Glavač and Glavač 2020; Suk and Rousseaux 2020; Tsunemi et al. 2020; Villa et al. 2020; Wang and Zhang 2020; Wesseling et al. 2020; Brodovitch et al. 2021; Campese et al. 2021; Dobrowolny et al. 2021; Gaurav et al. 2021; Lee and Woodruff 2021; Majeed et al. 2021; Mitchell et al. 2021; Pereira et al. 2021; Rusz et al. 2021; Soares Martins et al. 2021; Streubel-Gallasch et al. 2021; Van Der Zee et al. 2021; Yuan et al. 2021; Dejanovic et al. 2022; Gómez De San José et al. 2022; Hayes and Kalab 2022; Kocurova et al. 2022; Shepheard et al. 2022; Dang et al. 2023; Donini et al. 2023; Hansson et al. 2023; Kaštelan et al. 2023; Miteva et al. 2023; Monteiro et al. 2023; Prasath and Sumathi 2023; Vidovic et al. 2023; Zeng et al. 2023; Zhou et al. 2023; Arslan et al. 2024; Darabi et al. 2024; Li et al. 2024; Morrone Parfitt et al. 2024; Sechi and Sechi 2024; Zhang et al. 2024; Long et al. 2025; Mizielinska et al. 2025). Alterations in cardiolipin levels may also reflect mitochondrial integrity loss observed in multiple ALS models (Chaves-Filho et al. 2019). These findings imply that UA and cardiolipin levels in blood are the candidates of ALS prognostic biomarkers.

**Figure 3.**
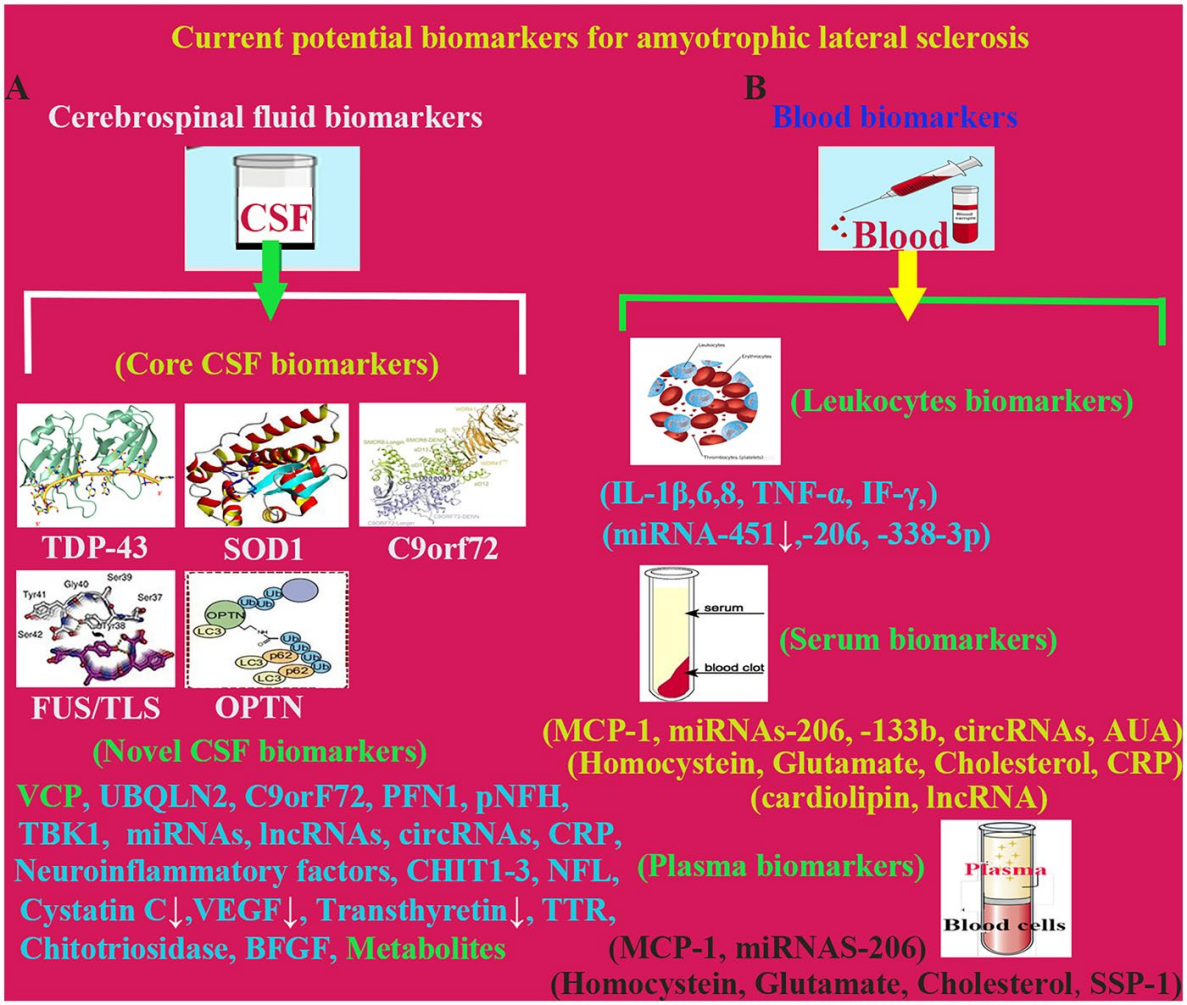
Current potential biomarkers for Amyotrophic lateral sclerosis. (A) Current potential biomarkers for AD in CSF, such as, core CSF biomarkers TDP-43, SOD1, C9orF72, FUS and OPTN, and novel CSF biomarkers. (B) Current potential biomarkers for ALS in blood, including leukocytes, serum and plasma biomarkers. Abbreviation: ALS, Amyotrophic lateral sclerosis; BFGF, Basic fibroblast growth factor; CHIT1-3, Chitinase-like protein 3; C9orF72, Chromosome 9 open reading frame 72; circRNAs, Circular RNAs; CRP, C-reactive protein; FUS/TLS, Fused in sarcoma/translated in liposarcoma; IF-γ, Interferons-γ; IL-1β,6,8, Interleukin-1β,6,8; lncRNAs, Long non-coding RNAs; MCP-1, Monocyte chemoattractant protein-1; miRNAs, microRNAs; NFL, Neurofilament light chain; OPTN, Optineurin; PFN1, Profilin 1; pNFH, Phosphorylated neurofilament heavy chain; SOD1, Superoxide dismutase 1; SPP-1 (Osteopontin), Secreted phosphoprotein-1; TBK1, TANK-binding kinase 1; TDP-43, Trans-reactive DNA-binding protein 43 kDa; TNF-α, Tumour necrosis factor-α; TTR, Transthyroxin; UA, Uric acid; UBQLN2, Ubiquilin 2; VCP, Valosin-containing protein; VEGF, Vascular endothelial growth factor.

CSF chitotriosidase (CHIT1) levels are observed to be elevated in ALS patients (Vidovic et al. 2023). CSF CHIT1 levels may provide additional prognostic evaluated value in ALS patients with short symptom history and challenging early diagnosis. Evidence indicates elevated CHIT1 likely reflects microglia/macrophage activation in the white matter of spinal cord. Based on these studied evidences, CHIT1 is positioned as a potential biomarker for ALS diagnosis and progression prediction (Yang et al. 2021) (Figure 3).

### The emerging biomarkers of ALS

Differences in microRNAs like miR-206 and lipid components within neuron-derived exosomes offer novel diagnostic clues for ALS. Current research is advancing the screening of ALS-specific non-coding RNA (ncRNA) biomarkers *via* RNA sequencing. Lipid metabolism dysregulation is closely linked to NDs, such as bis-monoacylglycerol phosphate (BMP) and glyco-sphingolipids like gangliosides monosialoganglioside GM1, disialoganglioside GD1a and glucocerebroside GlcCer as the most promising biomarkers. BMP is directly associated with lysosomal dysfunction in frontotemporal dementia and PD. Sphingolipids exhibit abnormal accumulation or metabolic disorders in multiple NDs including AD and ALS (Wei et al. 2023; Guo et al. 2024). Based on current evidences, miR-206, ncRNA as well as lipid components like BMP and glyco-sphingolipids are the most promising emerging biomarker of AD, PD and ALS.

Wearable devices monitor digital metrics like gait, finger tapping speed and integrate with MRI to assess brain atrophy, enabling more robust prognostic evaluations. This multimodal approach combines real-time motor function data with structural neuroimaging to track disease progression (Darabi et al. 2024). it is able to be emerging digital and imaging biomarkers for AD, PD and ALS, is a potential future studying direction of digital neuroimaging biomarker.

Integrating genomic data like polygenic risk scores, proteomic signatures and clinical parameters *via* machine learning allows for the development of more precise diagnostic and prognostic models. These frameworks enhance personalised medicine by identifying complex biomarker patterns across biological scales (Darabi et al. 2024). The artificial intelligence may develop the biomarkers of multi-omics and machine learning models of ALS.

ALS patients exhibit significant alterations in gut microbiome composition including reduced firmicutes abundance and increased cyanobacteria abundance at phylum level. Compared to HCs, elevated pathogenic genera such as bacteroides and parasutterella and decreased beneficial genera like faecalibacterium and bifidobacterium at genus level, lower α-diversity as well as functional pathways enriched in lipid and amino acid metabolism dysregulation, which suggests lipid metabolic dysfunction may contribute to disease progression. These studying evidences of gut microbiome composition hint that gut microbiome alteration is promising ALS risk predicting biomarkers.

Metabolic hormones, particularly insulin and amylin, are closely associated with the progression and functional status of ALS, potentially acting through mechanisms involving energy metabolism regulation, neuroinflammation and neuronal protection (Moțățăianu et al. 2024). Metabolic pathways represent promising therapeutic biomarker and targets, though longitudinal studies are needed to validate causal relationships between hormone levels and prognosis, as well as to explore the therapeutic potential of insulin and related molecules. From this perspective, metabolic hormone regulation is an important emerging valuable developing biomarker for diagnosis and therapy of ALS.

### The common characteristics of biomarkers in AD, PD and ALS

Biomarkers in AD, PD, and ALS often exhibit changes before the onset of clinical symptoms. For instance, CSF biomarkers like β-amyloid 42 may become abnormal before approximately decade years prior to the AD emergence of cognitive impairment. Similarly, gut microbiome alterations in PD can be detected around decade years before motor symptoms manifest. In ALS, neuronal-derived exosomal microRNAs like miR-206 show preclinical shifts. It implies that the common characteristics of β-amyloid 42 in AD, gut microbiome alterations in PD and miR-206 in ALS biomarkers are early detected prior of AD, PD and ALS onset, are potential important common characteristics for AD, PD and ALS biomarkers, which are the best candidate biomarkers for early diagnosis.

As diseases progress, biomarker levels or states evolve dynamically. In ALS, plasma NF-L concentrations rise progressively with motor neuron degeneration, while in AD and PD, pathological protein aggregates like Aβ plaques, tau tangles and α-synuclein inclusions and neuroinflammatory cytokines like TNF-α and IL-6 increase longitudinally, correlating with disease severity. For PD, dopamine transporter PET imaging demonstrates gradual decline in striatal uptake, reflecting dopaminergic neuron loss over time. The common characteristics of these AD, PD and ALS biomarkers are dynamic changes accompanying with the progression of AD, PD and ALS diseases, is the best biomarkers candidates of monitoring disease progression.

All three diseases exhibit abnormalities in peripheral blood inflammatory cytokines like IL-1β, IL-6, oxidative stress markers like 8-OHdG in urine and malondialdehyde in blood, which indicates the biomarkers of AD, PD and ALS involve widespread immune and metabolic dysregulation. Moreover, gut microbiome dysregulation such as reduced faecalibacterium and increased pathogenic genera affects lipid and amino acid metabolism in ALS and PD, which links gastrointestinal changes to central neurodegeneration. These study evidences indicate that the common characteristics of AD, PD and ALS biomarkers are not confined to nervous system but reflect multiple systemic pathophysiologies and exit lots of overlapped biomarkers among three NDs, which indicates that multiple biomarker application may be better choice for improving AD, PD and ALS accurately diagnosis.

The presence of protein misfolding-related biomarkers like Aβ, tau, α-synuclein and TDP-43 across all three diseases highlights shared disruption mechanism in protein homeostasis. In addition, neuroinflammation and oxidative stress markers like GFAP and S100B reveal common mechanism immune-mediated cellular lesion. Gut-brain axis dysfunction, retinal and skeletal muscle involvement, further broadens biomarkers resource such as cross-system interactions and reflecting integrated disease processes. In view of this, the common characteristics of AD, PD and ALS biomarker are that biomarkers mainly derived from comprehensive pathological mechanisms.

In summary, the common characteristics of AD, PD and ALS biomarkers are unified by their preclinical detectability, dynamic monitoring to disease progression, systemic pathological presentation and mechanistically characterise neurodegeneration. These characteristics collectively enable early diagnosis and intervention strategies, longitudinal progression monitoring as well as the development of therapeutic targeting shared pathways.

### Novel therapeutic approaches (biomarkers/targets intervention) for AD, PD and ALS

Current clinical therapies for AD primarily rely on acetylcholinesterase inhibitors and glutamate receptor antagonists to alleviate symptoms, there is not curative treatments to be finding yet up to now despite investing a significant amount of manpower and financial resources in AD research. Recent research (Fan et al. 2025) highlights that neuroprotective protein neurotrophic factor-alpha1/carboxypeptidase E (NF-alpha1/CPE) is closely associated with AD pathogenesis, though its specific mechanistic pathways remain unclear. Studies have confirmed a dose-dependent relationship between NF-α1/CPE expression levels, AD pathological features and cognitive function, which positions it as a promising therapeutic target.

Oestrogen replacement therapy has shown modest benefits in postmenopausal women with AD and PD, acting through mechanisms such as neuronal signalling modulation, tissue adaptive changes, gene expression regulation and antioxidant effects (Song et al. 2020). However, challenges including data selection bias, evolving diagnostic criteria, and difficulties in tracking hormonal therapy history necessitate further research on oestrogen administration methods and the specific roles of endocrine targets in neuroprotection (Song et al. 2020).

Dopamine replacement therapy remains the cornerstone of PD treatment but is often complicated by motor complications. Electroacupuncture (EA), a non-pharmacological intervention, has gained attention for its simplicity and low side-effect profile (Gai et al. 2021). However, whether EA combined with levodopa outperforms levodopa monotherapy remains inconclusive. A systematic meta-analysis evaluating response rates, Unified Parkinson’s Disease Rating Scale scores, Webster scores and biomarkers copper-zinc superoxide dismutase, membrane lipid peroxidation and dopamine levels aims to provide evidence-based insights into EA efficacy and safety (Liu et al. 2024). Non-carrier nanocapsules offer a novel drug delivery strategy by enabling the synergistic delivery of catalase and DA to brain parenchyma, leveraging enhanced dopaminergic signalling, reduced neuroinflammation and biocompatibility to address current therapeutic administration limitations (Liu et al. 2024).

Probiotic therapy has demonstrated promise in improving gut function in PD patients, significantly increasing defaecation frequency and constipation with acceptable side effects. However, regional biases in existing studies, the unclear effects of specific strains and the limited investigation of faecal short-chain fatty acid changes necessitate long-term studies to clarify probiotic impact on disease progression (Wieërs et al. 2019). Among them, lactobacillus- and bifidobacterium-based probiotics effectively relieve constipation with manageable side effects, while their disease-modifying potential requires validation yet (Hong et al. 2022).

ALS currently emerging therapy focuses on delaying progression *via* targeting pathological mechanisms. Among them, pharmacological interventions like riluzole (glutamate inhibition), edaravone (antioxidation), sodium phenylbutyrate/tauroursodeoxycholic acid (cell stress relief) and tofersen (SOD1 mRNA degradation) show modest efficacy but remain limited. Molecules like CLR01 (molecular tweezers) and green tea extract epigallocatechin-3-gallate reduce SOD1 aggregation, though motor function improvements in animal models are inconsistent (Al-Khayri et al. 2024). Neuroprotective agents such as vitamin E, curcumin and melatonin act *via* free radical scavenging and mitochondrial function enhancement.

In gene therapy, antisense oligonucleotides and clustered regularly interspaced short palindromic repeats targeting SOD1, C9ORF72 and other mutations are in clinical trials. In addition, induced pluripotent stem cell models facilitate mechanistic research of stem cells, thus, mesenchymal/neural stem cell transplantation holds promise for neuronal repair. Targeting neuroinflammation such as cannabinoids preserves the muscle function through protecting troponin activators, and combination therapies like nebivolol-donepezil are emerging therapy (Morimoto et al. 2019).

ALS research is hindered by unclear aetiology, lacks of early diagnostic tools and drug delivery/toxicity issues, suboptimal disease-modifying effects (Rizea et al. 2024). Future efforts should prioritise understanding pathogenic mechanisms, developing non-invasive biomarkers, accelerating gene editing/stem cell therapy and leveraging artificial intelligence for personalised treatment optimisation.

## Summary and prospect

The diagnosis of AD, PD and ALS relies primarily on clinical examinations, observed symptoms and tests to exclude mimicking disorders. Due to the multiplicity of mechanisms and biological processes underlying these diseases, no single biomarker currently achieves 100% specificity and diagnostic sensitivity for their diagnosis. However, ongoing advancements in innovative diagnostic tools and technologies hold promise for driving fundamental improvements and significant breakthroughs in both clinical practice and research for these conditions.

Expert clinical assessment remains central to diagnosis, as heterogeneous clinical presentations and multi-system involvement-such as need for interdisciplinary evaluation of cognitive and behavioural domains-pose significant challenges. In addition to clinical evaluation, electrophysiological assessments like electromyography and neuroimaging techniques like functional MRI and PET are increasingly critical for aiding early diagnosis, particularly in differentiating these diseases from related disorders. Biomarkers are urgently needed to enhance diagnostic accuracy, especially in the preclinical or early stages of these NDs. As disease-modifying therapies become available, the importance of timely and accurate diagnosis will only grow. Accessible, cost-effective and reliable diagnostic biomarkers have potential to transform clinical applications across diverse settings.

Beyond diagnosis, biomarkers are essential for providing progression assessment, prognostic insights, optimising treatment strategies, tailoring supportive care and identifying eligible candidates for clinical trials. Thus, the development of robust biomarkers for AD, PD and ALS represents a critical unmet need in neurology, with direct implications for improving patient outcomes and advancing therapeutic discovery.
